# Combined approaches from physics, statistics, and computer science for *ab initio* protein structure prediction: *ex unitate vires* (unity is strength)?

**DOI:** 10.12688/f1000research.14870.1

**Published:** 2018-07-24

**Authors:** Marc Delarue, Patrice Koehl

**Affiliations:** 1 Unité Dynamique Structurale des Macromolécules, Institut Pasteur, and UMR 3528 du CNRS, Paris, France; 2 Department of Computer Science, Genome Center, University of California, Davis, Davis, California, USA

**Keywords:** Protein structure prediction, template-free prediction, secondary structure prediction, co-variation

## Abstract

Connecting the dots among the amino acid sequence of a protein, its structure, and its function remains a central theme in molecular biology, as it would have many applications in the treatment of illnesses related to misfolding or protein instability. As a result of high-throughput sequencing methods, biologists currently live in a protein sequence-rich world. However, our knowledge of protein structure based on experimental data remains comparatively limited. As a consequence, protein structure prediction has established itself as a very active field of research to fill in this gap. This field, once thought to be reserved for theoretical biophysicists, is constantly reinventing itself, borrowing ideas informed by an ever-increasing assembly of scientific domains, from biology, chemistry, (statistical) physics, mathematics, computer science, statistics, bioinformatics, and more recently data sciences. We review the recent progress arising from this integration of knowledge, from the development of specific computer architecture to allow for longer timescales in physics-based simulations of protein folding to the recent advances in predicting contacts in proteins based on detection of coevolution using very large data sets of aligned protein sequences.

## Introduction

Proteins are essential macromolecular biomolecules to all organisms, as they participate in nearly all processes within cells, thereby sustaining life. They catalyze most biochemical reactions and are involved in maintaining the integrity of the genomic information. They control and perform self-replication, transport molecules (including nutrients) in and out of the cell, play a role in immune responses, and control cell cycles; those are only a small subset of the activities associated with proteins within a cell. The amino acid sequence of a protein is defined by the nucleotide sequence of its gene. This relationship is well known and captured by the genetic code. The function of a protein then is defined by the geometry adopted by the corresponding chain of amino acids, the so-called tertiary structure of the protein. However, understanding the relationships between the sequence and structure of a protein, the second piece in the puzzle of understanding how proteins function, remains elusive. This review is focused on the computational approaches developed to unravel these relationships.

Although much progress has been achieved in connecting protein sequence to their function, there remains a high proportion of genes with unknown function, especially in bacteriophages and, even more dramatically, in archaeal viruses, in which 75 to 90% of their genomes remain largely unknown ^1^. Determination of the function of a protein experimentally is a highly resource-intensive process. As a consequence, much hope is put into alternate approaches based on computational methods. Sequence analysis is usually the first step, as significant sequence similarity is still the most reliable way of inferring function. There are, however, some exceptions to this inference, from proteins with nearly identical sequences but different functions ^2^ to proteins with different sequences but similar functions ^3^. When sequence is uninformative when it comes to predicting function or when there are no detectable homologues in the databases of annotated protein sequences, structure can often provide further insight. Therefore, significant efforts are put into predicting function from structure ^4^. These efforts rely obviously on the availability of structural information.

Our current knowledge of protein structure comes mostly from decades of experimental studies, using X-ray crystallography, nuclear magnetic resonance spectroscopy, or more recently cryo-electron microscopy. The first protein structures to be solved were those of hemoglobin and myoglobin more than 50 years ago ^5– 7^. As of April 2018, there are close to 140,000 protein structures in the database of biological macromolecular structures ( http://www.rcsb.org). However, this number drops to about 50,000 if one keeps sequences with less than 90% sequence identity ( http://www.rcsb.org/pdb/statistics/clusterStatistics.do). This number is obviously small compared with the number of existing proteins. Although the incentive to significantly increase the number of experimentally defined protein structures is evident (for instance, for drug-design purposes), how to achieve such a drastic increase is less clear, as the cost (mostly in man-months) to solve a new protein structure is very high. Unfortunately, this high cost led to the ending of the Protein Structure Initiative by the National Institutes of Health in 2015, long before it had reached its goal. As a reminder, the main ideas behind this initiative were to progressively document a full library of possible natural protein folds and to use the structure of new proteins to annotate them functionally ^4, 8^.

There is hope for an alternate solution to complement the experimental efforts to characterize the protein structure space. From the seminal work of Anfinsen ^9^, we know that the sequence fully determines the three-dimensional structure of a protein. In addition, the information on protein sequences is growing exponentially: as of April 2018, there were more than 557,000 protein sequences deposited in SwissProt-Uniprot version 2018-03, the fully annotated repository of protein sequences (note that these form a small subset of all known proteins; the RefSeq database of non-redundant proteins currently contains more than 110 million sequences). As a consequence, there is a lot of effort put into predicting the structure of a protein directly from the knowledge of its sequence (and its relatives). This has been referred to as one of the “holy grails” in molecular biology, also called the protein structure prediction problem. This problem has been around for decades ^10, 11^ but clearly remains a very active area of research. This is illustrated, for example, by the following: (i) a PubMed search with the keywords “protein structure prediction” finds more than 4,000 hits since 2014; (ii) this field has its own dedicated biannual conference, the Critical Assessment of Structure Prediction (CASP) meetings ^12– 14^; (iii) it has its own section in F1000; and (iv) it is even the topic of a popular video game ^15, 16^. In this article, we review the latest advances related to solving this problem and put a special focus on highlighting the specific scientific fields that are involved in those advances.

Protein structure prediction is in fact part of a larger problem, the protein-folding problem. The latter can be seen as an investigation over two questions: (i) understanding the kinetic folding mechanism, namely unraveling the temporal sequence of events describing how a polypeptide chain finds its way from an unfolded structure into a compact, globular conformation in a seemingly unreasonably fast time given the number of possible conformations it may adopt (the Levinthal paradox ^17, 18^), and (ii) understanding the physical folding code, namely deciphering how the physicochemical properties of the amino acids along the polypeptide chain that form a protein sequence are uniquely responsible for the tertiary structure of a protein and its stability. We focus on the second question and recent computer-based solutions to this problem. We note first that most successful structure prediction algorithms rely on the assumption that similar sequences lead to similar structures ^19^. This has led to the development of comparative modeling techniques, which have seen considerable improvements over the years, as reported in the overview of the most recent CASP meeting ^14, 20^. Based on those reports, homology modeling methods are estimated to produce reasonable atomic models provided that the alignment has a sequence identity above 50%, with high coverage (>80%). We will not cover this topic that has been reviewed extensively elsewhere ^21– 23^. Instead, we review results in the more challenging problem of template-free ( *ab initio*) modeling. More specifically, we limit our presentation to recent techniques that have been developed with physics-based and machine-learning perspectives. We focus on free energy-based methods, secondary structure prediction, and contact predictions. We provide our own perspective on recent progress in each of these topics; recent technical reviews can be found elsewhere ^10, 24– 35^.

## Physics-based approaches to protein structure prediction

The stability of a protein structure is defined by an interplay of multiple factors, from the local geometry imposed by structural chemistry (bond lengths, bond angles, and dihedral angle preferences) to close-range physical interactions that govern the structures of all liquids and solids. Among those, van der Waals interactions lead to tight packing in the protein core, and electrostatic interactions, such as salt bridges and hydrogen bonds, define short- or long-range couplings that underlie, for example, allostery ^36, 37^ and active transport ^38, 39^. In addition, a protein interacts with its environment, namely water, which creates a polar environment, forcing hydrophobic residues to pack into a hydrophobic core, and ions (Na ^+^, Cl ^−^, Mg ^2+^, etc.), which interact with charged residues at the surface of the protein. All of those interactions are captured by semi-empirical “force fields”, with different levels of approximation, from implicit or explicit solvent, presence or absence of terms accounting for polarization, to complete treatment of classic electrostatics ^40, 41^. Combined with sampling methods such as molecular dynamics (MD) or Monte Carlo (MC) simulations, these force fields can be used to generate ensembles of conformations for any protein structure, from which the native tertiary structure hopefully can be selected ^42^. The input to such sampling techniques can be an initial guess for the conformation of the protein, obtained, for example, from comparative modeling; this procedure is referred to as model refinement. Although progress associated with this problem is always optimistically conveyed in the reports from the successive CASP meetings (for example, ^43^), there is a growing consensus that the current techniques have reached a plateau and that a combination of better force fields and improved sampling is needed for further improvements ^44, 45^.

The real challenge is to start from any random conformation for the protein and hopefully induce, through *in silico* molecular simulation in the computer, the folding to the native structure of the protein. These simulations have the advantage of directly sampling the free energy surface spanned by the protein. In addition, if successful, they solve the structure prediction problem and provide kinetic and thermodynamic information about folding. As the size of the conformation space is overwhelmingly large, the general understanding in the structure prediction community for a long time was that such an approach would be limited to predicting the structure and folding of small peptides ^46^ unless it could be supplemented with additional experimental ^47– 49^ or data-based ^50– 52^ information. This limited optimism was fortunately proven wrong on at least a few occasions, mostly as a consequence of changes in computing power and in how computing is performed. One example is Folding@home ^53, 54^, developed by Vijay Pande at Stanford University, in which computer users all over the world donate their idle computer time to perform the physical molecular simulations required for protein folding. Using this form of “social distributed computing”, the Pande group demonstrated that molecular dynamics can generate accurate protein-folding rates ^55^. More recent results with this distributed computing approach include the analyses over “long” (over the millisecond) time ranges of the transitions between inactive and active forms of G-protein-coupled receptors (GPCRs) (proteins with 500 amino acids) ^56^ and modeling of the activation pathways of Src kinases (250 residues included in the simulations) ^57^. The success of Folding@home has led the computational biology community nowadays to expect “routine” simulations of protein dynamics in the millisecond timescale and longer. We also note an interesting trend within the field of MD simulations in terms of methods development. Such simulations request constant development of the algorithms implemented for performing the dynamics; the community has responded well to this need, and new versions of the software packages appear regularly; see, for example, the recent developments around OpenMM ^58^, the software package behind Folding@home. These new software packages push the limits of what can be achieved with those simulations, enabling the study of larger and more complex systems, which in turn foster the development of new methods for analyzing the results. Indeed, these large simulations generate massive amounts of data, raising new challenges in data sciences. Sophisticated statistical machineries have been proposed to analyze those data, such as the Markov state models ^59– 61^, which are constantly updated ^62^, to the point of switching “from [being] an Art to [becoming] a Science”, paraphrasing a recent review by Husic and Pande ^63^. Interestingly, the specificity of the data generated by molecular simulations has led to the natural adaptation of machine-learning techniques to support their analyses; for a recent review on this topic, see Mittal and Shukla ^64^.

An alternate approach to distributing computing is to adapt the computing hardware to the equations and the force field implemented in molecular simulations. The Anton computer from DE Shaw Research, which was custom-designed for molecular simulations ^65, 66^, gives at least one order of magnitude better performance than conventional computers ^67^. Using this new technology, this group was able to study, for example, the dynamics in human ubiquitin in the picosecond to millisecond time range using unbiased dynamics, revealing that conformations visited in the simulations are very similar to those found in crystal structures of ubiquitin and detecting correlated motions in the protein that are consistent with experimental observations ^68^. Of equal significance, simulations performed on Anton are helping to identify the structural origins of slow diffusion during protein folding ^69, 70^.

Finally, it should be mentioned that advances in computing models (either cloud-based or with a specific architecture) are not the only options to improve simulations of protein folding. Dill *et al*. recently illustrated that the addition of some semi-reliable external information to the potential that steers the molecular simulation enables accurate prediction of the native conformations for small protein structures within the context of CASP ^52, 71^. This information is provided in the form of binary residue contacts deduced from the protein sequence itself. This will be discussed in more detail below.

## Secondary structure prediction: significant improvements from machine learning

Ultimately, the structure of a protein is defined by the laws of chemistry and physics. In the section above, we described attempts to derive a physics-based solution to the structure prediction problem; our tone was meant to be positive and optimistic, but we cannot deny that those attempts have their limitations. At the same time, the structure prediction community is exploring methods outside the realm of physics-based first-principles equations. Secondary structure prediction, a subproblem of protein structure prediction, is a good example of how methodologies in this whole field have evolved over the last 50 years.

Here, we are concerned with the prediction of the local conformation of the backbone of a protein, namely its organization into helices, strands, and coils (that is, loosely structured regions). As discussed in recent reviews of secondary structure prediction, this problem is important in its own right, as it impacts many areas of structural bioinformatics and functional genomics ^25, 28^. Linus Pauling can be considered the father of this field, as he correctly predicted the presence of helices and strands as stable substructures in proteins ^72, 73^. In the nearly 80 years that followed his intuition, secondary structure prediction has struggled to come up with a definite solution through at least three phases, which Burkhard Rost referred to as generations ^74^. Briefly, the starting idea was to derive statistical preferences for amino acids to be within a specific secondary structure based on known protein structures and use those preferences for predictions. This inference, also called the inverse problem, proved harder than expected. Chou and Fasman, for example, derived empirical rules for both helices and strands ^75^. However, refining those rules proved to be a game with moving targets, and the method remained limited in scope. The second generation of methods focused on either the definition of the propensities themselves, making them locally context-dependent to include neighborhood effects ^76^, or how to derive the rules for inference, relying more and more on learning those rules from the data instead of enforcing a specific model. The latter led to the introduction of neural networks in the field ^77^. This second generation unfortunately led to only modest improvement. However, once the door was open to learning from the data, it was then only natural to increase the amount and diversity of the data that are included to generate better models. The most obvious choice was to introduce information from homologous sequences ^78^, which led to the third generation of secondary structure prediction methods, including the significantly improved neural network solutions implemented in PhD ^79^ and PSIPRED ^80^. Those methods were introduced in the 1990s. Since then, we have seen the development of more and more sophisticated machine-learning methods with more involved neural networks, such as the recent use of deep neural networks ^81^ and deep convolutional neural fields ^82^ for secondary structure prediction (for a comprehensive review of the different types of networks and machine-learning techniques that have been applied to solve the secondary structure prediction problem, see 28). This recently led Yang *et al*. to ask whether we are in “the final stretch” for protein structure prediction ^25^. Although we appreciate their optimism, we raise a small word of caution. The goal of machine-learning methods is to identify recurrent patterns in data that can be used to solve the inference problem: that is, connecting features (here amino acid sequences) to underlying variables (here secondary structure conformation). Optimizing a model to a generalized inference model usually prevents prediction of atypical behavior. However, we note that this is a problem for machine learning in general.

## Predicting contacts in proteins: (statistical) physics or machine learning?

Protein structures are more conserved than their sequences. It is therefore legitimate to expect that correlated mutations occur between contacting residues: in the event that one residue of such an interacting pair mutates, the effect of this mutation is likely to be accommodated by a corresponding mutation of the contacting residue. This was discussed in detail in the recent analysis of coevolution between residues by Baker *et al*. ^83^. This basic idea implies that if we can detect co-variation in sequences, we should be able to predict contacts in protein.

The possibility to detect co-variations has significantly increased with the increase in the number of protein sequences that are available. The related search for an inference between those co-variations and actual contacts in protein has been the focus of decades of research, and recent breakthroughs are highlighted below. We will limit our presentation to general concepts (especially the mean-field approach) and refer readers to excellent recent reviews for more technical presentations ^34, 35, 84^.

Let us consider a multiple sequence alignment (MSA) for a protein family *P*. This MSA can be considered as a table *T*, consisting of *L* rows corresponding to the *L* proteins included in the MSA and of *M* columns, where *M* is the length of the proteins. The value *T(l,m)* at row *l* and column *m* is the type of amino acid observed at the *m*-th position of protein *l*. *T(l,m)* is assumed to be represented by an integer number in *[1,q]*, where *q = 21*, representing the 20 amino acids and a gap. The information in this MSA is first summarized in terms of frequencies,

$$f_{i}(a)=\frac{1}{L}\sum_{l=1}^{L}\delta_{a,T(l,i)}\;f_{\text{ij}}(a,b)=\frac{1}{L}\sum_{l=1}^{L}\delta_{a,T(l,i)}\delta_{b,T(l,j)},\;\left(1\right)$$

with *i* and *j* in *[1,M]* and *a* and *b* in *[1,q]* and *δ* the Dirac function such that *δ _xy_ = 1* if *x = y*, and 0 otherwise. In practice, weights and pseudo-counts are introduced to account for possible biases in the distribution of sequences in the MSA ^85^; we ignore them here for the sake of simplicity. In these definitions, *f _i_(a)* is the frequency of the occurrence of amino acid *a* at column *i* in the MSA, and *f _ij_(a,b)* is the frequency of co-occurrence of amino acid types *a* and *b* at columns *i* and *j*. If the two columns *i* and *j* are independent, the joint distribution *f _ij_(a,b)* would simply be equal to the product *f _i_(a) f _j_(b)*. Deviations from this independence can be either quantified by using a covariance

$$C_{ij}(a,b)=f_{ij}(a,b)-f_{i}(a)f_{j}(b)\;\left(2\right)$$

or summarized by computing the mutual information between columns *i* and *j* ^86^

$$M_{ij}=\sum f_{ij}(a,b)\text{ln}\frac{f_{ij}(a,b)}{f_{i}(a)f_{j}(b)}.\;\left(3\right)$$

It is tempting to infer spatial proximity from those covariance or mutual information measures (or any variation of). This idea was pursued as early as in the 1980s for predicting virus functions ^87^ and in the 1990s to detect contacts in proteins ^88, 89^. However, it met with little success, for a reason that can be described as follows ^30, 85^: when a residue *i* is in contact with a residue *j*, and *j* is in contact with a residue *k*, then *i* and *k* will exhibit correlation, even if they are not in contact. Correct analyses of the observed co-variations (computed by using equation 1 and equation 2) require that direct interactions be distinguished from possible indirect correlations. Currently, two approaches are being developed to disentangle those direct and indirect variations and infer contacts from observed co-variations: those that construct a statistical model for full-length protein sequences by using methods from statistical physics and those that learn this model from the data by using machine-learning techniques.

Among the statistical methods developed for co-variation analyses, sequence-based probabilistic formalisms have been proposed as early as 2002 ^90^, followed by message-passing algorithms ^91^, mean-field methods ^85^, and Gaussian ^92^ or pseudo-likelihood ^93, 94^ approximations. More precise methods, based on adaptive cluster expansion ^95^ or Boltzmann learning using MC sampling ^96, 97^, have been proposed recently. In the following, we briefly review the mean-field methods.

From a statistical physics point of view, the protein sequence space can be equipped with a spin-glass Hamiltonian model such that the Hamiltonian of a sequence *S* of length *M* is given by

$$H(S)=-\sum_{i=1}^{M-1}\sum_{j=i+1}^{M}J_{ij}(s_{i},s_{j})-\sum_{i=1}^{M}h_{i}(s_{i}),\;\left(4\right)$$

where *s _i_* is the amino acid type at position *i* in *S*, *h _i_(s _i_)* are single-site “fields”, and *J _ij_(s _i_,s _j_)* are pair-site “couplings” between *i* and *j*. When the size of the alphabet of amino acid types is 2, equation 4 corresponds to an Ising model. In the more general case of an alphabet of size 21, equation 4 is referred to as a Potts Hamiltonian model. Given this model, the probability of observing a sequence *S* follows a Boltzmann distribution,

$$P(S)=\frac{e^{-\beta H(S)}}{\sum_{C}e^{-\beta H(C)}},\;\left(5\right)$$

where *β = 1/kT* is a normalization parameter, and the sum at the denominator runs over all sequences *C* of length *M*. Note that this denominator is the partition function *Z* over the sequence space, from which a free energy can be derived:

βF=−log⁡(Z).(6)

The knowledge of *Z* (or *F*) is enough to fully describe the thermodynamics of the system. In particular, the mean state at position *i* and the correlation between positions *i* and *j* can be computed as

$$<s_{i}>\text{=}\frac{1}{Z}\frac{dZ}{dh_{i}}\;<s_{i}s_{j}>\text{=}\frac{1}{Z}\frac{d^{2}Z}{dh_{i}dh_{j}}.\;\left(7\right)$$

The parameters *h _i_(s _i_)* and *J _ij_(s _i_,s _j_)* need to be adjusted such that these model-based state and correlation values are consistent with the observed values given in equation 1. This is the basic concept behind the direct coupling analysis (DCA) ^98, 99^. Several versions of DCA, differing in the method used to compute *h _i_(s _i_)* and *J _ij_(s _i_,s _j_)*, have been proposed. In the mean-field approximation, there is a simple relationship between *J _ij_* and the covariance *C _ij_* defined in equation 2:

$$J_{ij}(a,b)=-{\left(C^{-1}\right)}_{ij}(a,b).\;\left(8\right)$$

Recent versions of DCA typically reach accurate prediction of about 85% true positives and only 15% false positives at the 8 Å distance (typically used as the definition of “contact” in DCA studies) ^35^. This success has led to DCA being used outside of the protein structure prediction problem, from the prediction of the structure of protein complexes ^34, 100^ and protein conformational transitions ^96, 101^, RNA structure prediction ^102– 104^, and prediction of ordered states for disordered proteins ^105^ to the prediction of mutation effects in proteins ^106^.

The Potts Hamiltonian model for defining the stability of a protein sequence summarizes all forms of interactions between residues into two simple sets of parameters: single-site and pair-sites. As the information content of an MSA goes beyond those simple interactions, it was natural to see attempts to combine such additional information to co-variation measures by using machine-learning techniques, much akin to what has been done for secondary structure prediction (see above). In the popular MetaPSICOV ^107^ approach, for example, a first-stage classifier is trained to predict whether residues are in contact by considering 672 input features for each pair of positions *i* and *j* in an MSA. Three windows are defined: one nine-residue window centered at *i*, one nine-residue window centered at *j*, and a central five-residue window centered at *(i+j)*/2. Each of those 23 positions then is characterized by its amino acid composition in the MSA (21 values), followed by information on secondary structures and solvent accessibility, both predicted from the MSA itself. These position-specific features then are complemented with coevolution information, such as mutual information (computed with equation 3) and DCA-predicted contact information, as computed with equation 8. The complete set of 672 features then is used to predict whether the positions *i* and *j* define a contact, using different cutoff values for defining such a contact. The outputs of the first-stage classifier then are used as input to a second-stage classifier, again combining information over windows around the residues under scrutiny. MetaPSICOV is a hybrid method: it uses both the results of the statistical physics DCA method and additional information derived from the MSA to predict contact. It has been shown to achieve higher accuracy than DCA alone ^107^. The success of MetaPSICOV is not isolated. It is interesting, for example, that out of the 23 methods that have been used for contact predictions in CASP12, at least 21 are clearly based on machine learning, using different versions of deep learning methods or combinations of such methods ^108^. Those methods were reported to be strikingly successful, and precisions above 90% were achieved by the best predictors in more than half the targets in CASP12, a result considered to be a highly significant improvement compared with the results obtained during CASP11 ^108^. In this respect, it will be particularly important to watch the result of CASP13 (2018), which will fully integrate mature contact prediction methods ( http://predictioncenter.org).

## Conclusions

Is the protein structure prediction problem “solved”? In the past, many have claimed that to be the case or have at least described progress and remaining challenges ^109, 110^. In line with a recent review by Dill and MacCallum ^10^, it is unclear to us whether this question remains relevant, given the diversity associated with structure prediction, both in terms of the methods that have been developed as attempts to solve it and in terms of their applications, from predicting the structures of small globular proteins, of membrane proteins, and the conformational space accessible to intrinsically disordered proteins. We also note the difficulties that come with assessing what “success” means, and this is illustrated by the need to have a series of conferences dedicated to that problem, the CASP conferences ^12^.

The prediction of contacts in proteins, described in this review, is a good example that highlights the constant evolution of the field of protein structure prediction. The concept of correlating co-variations between residues in sequence alignments to spatial proximity was well known for RNA structure prediction. As highlighted above, its application to proteins was delayed because of the difficulties of dealing with indirect variations. Independent applications of statistical physics methods and machine-learning methods have, to some extent, solved those difficulties, opening the door to predicting geometric contacts in proteins from sequence only. This progress leads, however, to the next challenge: using those contacts to predict the overall geometry, that is, the structure of a protein. This is by no means an easy problem, as those contacts are usually given as binary, noisy information. Significant methodological developments are expected to solve this new challenge, which in turn will lead to yet another challenge.

The protein structure prediction problem intrinsically relates to basic science. However, it has led to important methodological and technical developments, from computer hardware for physics-based simulations to statistical methods and new machine-learning technologies. In practice, in biology, it will have an impact on many diseases related to protein misfolding, from neurodegenerative diseases to diabetes through the prediction of stability of mutants (EVmutation website: http://marks.hms.harvard.edu) and perhaps even to personalized medicine.

Attempts to solve the protein structure prediction problem have involved scientists from a large panel of disciplines, including biology, physics, statistics, and computer science. Even if the divide between physics-based approaches and data-driven methods still exists, this divide has been constructive and not disruptive. Data scientists involved in structure prediction are regularly using results from physics-based models, and reciprocally physicists are adding more and more data coming from data mining in their simulations. Therefore, the question we have raised in the title of this review may be as irrelevant as the question of whether the problem is solved: the future of protein structure prediction is interdisciplinary and will remain so.

## Abbreviations

CASP, Critical Assessment of Structure Prediction; DCA, direct coupling analysis; MC, Monte Carlo; MD, molecular dynamics; MSA, multiple sequence alignment
